# Letermovir does not affect long-term polyclonal immune reconstitution after allogeneic hematopoietic stem cell transplantation with ATG-based GvHD prophylaxis

**DOI:** 10.3389/fimmu.2026.1790563

**Published:** 2026-05-21

**Authors:** Conrad Weidt, Adela Maria Neagoie, Maral Saadati, Daniel Fürst, Verena Wais, Christian Sinzger, Katrin Strauss, Katharina Göhring, Jacqueline Schnell, Michaela Feuring, Frank Stegelmann, Axel Benner, Detlef Michel, Thomas Stamminger, Hartmut Döhner, Donald Bunjes, Elisa Sala

**Affiliations:** 1 Department of Internal Medicine III, Hematology and Oncology, University Hospital Ulm, Ulm, Germany; 2 Division of Biostatistics, German Cancer Research Center, Heidelberg, Germany; 3 Department of Transplantation Immunology, Institute for Clinical Transfusion Medicine and Immunogenetics Ulm, German Red Cross Blood Transfusion Service, Baden Wuerttemberg - Hessen, University Hospital Ulm, Ulm, Germany; 4 Institute for Virology, University Hospital Ulm, Ulm, Germany

**Keywords:** allogeneic stem cell transplantation, human cytomegalovirus, immune reconstitution, letermovir, pre-emptive treatment

## Abstract

**Introduction:**

Letermovir (LET) is an effective prophylaxis for human cytomegalovirus (HCMV) reactivations in HCMV-seropositive patients after allogeneic stem cell transplantation (allo-SCT). HCMV promotes polyfunctional T-cell responses, leading to HCMV-specific immune reconstitution (IR), thus contributing to polyclonal IR.

**Methods:**

We retrospectively analyzed HCMV-seropositive patients undergoing allo-SCT with ATG-based GvHD prophylaxis to assess the impact of LET on timing and quality of polyclonal IR. Two cohorts were identified: a pre-emptive treatment (PET) historical cohort transplanted without LET prophylaxis and a LET cohort receiving LET until at least day +100. All patients underwent weekly HCMV-DNA monitoring during the first 100 days post-allo-SCT. Immune monitoring was performed by flow cytometry, quantifying CD3+CD4+, CD3+CD8+ T-cells, CD19+ B-cells, and CD56+CD16+ NK-cells monthly during the first year after transplantation.

**Results:**

A total of 276 HCMV-seropositive patients were analyzed, 99 (36%) in the LET cohort and 177 (64%) in the PET cohort. LET significantly reduced the incidence of clinically significant HCMV infections during the first 100 days [28.3% in the LET cohort vs. 68.4% in the PET cohort (p<0.001)]. The cumulative incidence of IR 18 months after allo-SCT was comparable in the two groups [24% in the LET cohort vs. 27% in the PET cohort (p=0.393)]. These data were supported by multivariable analysis (HR 1.2, 95% CI 0.74–1.95, p = 0.463). Distinct immune dynamics were observed in the LET cohort, including lower early CD8+ T-cell counts and earlier NK-cell expansion peak.

**Discussion:**

Despite these differences, LET does not appear to influence the long-term incidence and composition of IR.

## 1 Introduction

Allogeneic hematopoietic stem cell transplantation (allo-SCT) is the most established curative approach for several high-risk hematologic malignancies (1). Nevertheless, despite the introduction of reduced-intensity conditioning regimens and improvements in supportive care, allo-SCT still carries a considerable risk of non-relapse mortality (NRM). Long-term follow-up studies indicate that NRM accounts for approximately 20% of overall mortality within the first 3 years after transplantation (2, 3). According to data from the Center for International Blood and Marrow Transplant Research (CIBMTR) infections are the second leading cause of NRM, contributing to 16% of deaths occurring before day 100 and 14% thereafter (4). Among these, viral infections have a particularly high incidence due to impaired T-cell function during the first months after allo-SCT, with human cytomegalovirus (HCMV) representing one of the most common and clinically significant pathogens (5, 6). Transplant recipients who have positive HCMV serology are at increased risk for clinically significant HCMV infections, end organ disease and both early and late NRM (7–9). The clinical course of HCMV infection after allo-SCT and its impact on patient’s outcome is not determined solely by viral replication, but rather by the interplay between viral dynamics and the pace and quality of immune reconstitution (IR) (8, 10). In particular, the recovery of HCMV-specific T-cell and natural killer (NK) cell responses play a pivotal role in preventing persistent viremia and progression to overt disease (11–13). In this setting, HCMV itself actively shapes IR: early viral reactivations leave a long-lasting imprint on the T-cell compartment, characterized by expansion of HCMV-specific CD8+ T cells, higher overall CD8+ counts, and a contraction of the T-cell receptor repertoire diversity (14, 15). Moreover, the kinetics and magnitude of HCMV viremia have been shown to differentially influence long-term outcomes, including mortality, relapse risk, and the overall quality of immune recovery (16). In 2017 letermovir (LET), an antiviral agent that inhibits HCMV replication by binding to components of the terminase complex (UL51, UL56, or both) (17), was approved for the prophylaxis of HCMV after allo-SCT in HCMV-seropositive recipients, leading to a significant decrease in the incidence of clinically significant HCMV infections, particularly within the first 200 days post allo-SCT (18, 19). Since the first 6 months after allo-SCT are critical for the establishment of both HCMV-specific and polyclonal IR—and given the active role of HCMV itself in shaping this process— the investigation of immune recovery in the era of LET prophylaxis is of crucial importance. Recent studies have shown that LET, while effectively preventing clinically significant early reactivations, may delay the reconstitution of HCMV-specific T-cell immunity, particularly CD8+ T cells, with recovery occurring later in the post-transplant course (20). This phenomenon has been associated with an increased risk of late-onset reactivations after drug discontinuation (18, 19). Considering the polyclonal IR and its interplay with the development of HCMV-specific IR, to our knowledge only one study has so far investigated the dynamics and potential paradigm shifts in the setting of polyclonal IR under LET prophylaxis (21). Orofino and colleagues (21) pointed out that with a post-transplant cyclophosphamide (PT-Cy)- based, calcineurin inhibitor- (CNI) free, graft versus host disease (GvHD) prophylaxis the use of LET in the first 100 days after allo-SCT produced a transient delay in T-cell reconstitution with an improvement of B-cell and NK-cells recovery, respectively by day +90 and +180 after transplantation. These findings are particularly relevant as PT-Cy generates a distinctive immunological milieu, with specific dynamics of T- and NK-cell recovery that differ from other GvHD prophylaxis platforms. At the same time, the impact of LET on polyclonal IR in patients receiving anti-thymocyte globulin (ATG) has been less extensively characterized. Furthermore, according to a recent published meta-analysis the risk of clinically significant HCMV infections was reported to be lower following the use of PT-Cy as compared to ATG, except for the haploidentical allo-SCT setting (22). Consequently, addressing this knowledge gap is crucial, given the widespread use of ATG in allo-SCT as a backbone for the GvHD prophylaxis (23) and its effects on both HCMV-specific and polyclonal IR, as delayed IR not only predisposes patients to late-onset clinically significant HCMV infections but also to other opportunistic infections, thus impacting on overall transplant outcomes. The present study aims to investigate the impact of LET prophylaxis on the development and timing of polyclonal IR in HCMV-seropositive patients undergoing allo-SCT after a GvHD prophylaxis backbone mainly based on ATG and CNI. By comparing patients receiving LET prophylaxis to those managed with pre-emptive therapy (PET), we seek to delineate the effects of antiviral prophylaxis on the reconstitution of key immune subsets, including T cells (CD4+ and CD8+), B cells, and NK cells. Understanding these interactions will inform strategies to optimize post-transplant care, balancing effective viral prophylaxis with the promotion of robust and timely polyclonal IR.

## 2 Patients and methods

### 2.1 Data collection and eligibility criteria

We performed a retrospective study of consecutive patients undergoing allo-SCT for various high-risk hematological diseases between 2013 and 2023 at the Bone Marrow Transplantation Unit of the Ulm University Hospital (Germany). The study was approved by the local ethics committee of the University of Ulm (Application Nr 356/23). All participants provided written informed consent for the pseudo-anonymous data registration and analysis in scientific context. Inclusion criteria were: (1) confirmed diagnosis of hematological disease, malignant or non-malignant, with an indication for allo-SCT; (2) adult age (≥ 18 years); (3) allo-SCT performed between 2013 and 2023, to ensure a relatively homogeneous population with respect to conditioning regimens and supportive care; (4) HCMV seropositivity at the time of transplantation; (5) ATG as backbone for the GvHD prophylaxis. To assess the effects of LET on polyclonal IR, we defined two cohorts: (a) the LET cohort, including patients transplanted from 2018 to 2023 and undergoing LET prophylaxis for HCMV reactivations at least during the first 100 days after allo-SCT; (b) the control cohort, consisting of HCMV-seropositive patients, who underwent allo-SCT before the approval of LET and were monitored for viral reactivations during the post-transplant follow-up, receiving PET if required (PET cohort).

### 2.2 HCMV monitoring, prophylaxis and treatment

All patients underwent monitoring for HCMV-DNA in peripheral blood using a PCR assay that was calibrated to the international HCMV standard and showed high analytical performance (intra-assay CV 1.2%, inter-assay CV 1.0%, accuracy 99%, sensitivity and specificity 100%) with a quantification range of 2 × 10^2^–2 × 10^7^ IU/ml (R^2^ = 0.99). Results were reported in IU/ml Treatments and HCMV-associated complications were collected from patient records. The monitoring was planned once a week for the first 100 days after allo-SCT. An intensification at twice weekly was performed in case of HCMV positivity or during PET treatment. Afterwards, patients underwent HCMV monitoring at regular follow-up appointments or upon the development of HCMV-related symptoms until the occurrence of polyclonal IR.

In the LET cohort, LET was administered at a dose of 480 mg/day, or 240 mg/day in patients receiving cyclosporine A (CsA) as part of the GvHD prophylaxis. LET was discontinued on day +100 after allo-SCT, except in high-risk patients (e.g., those with active GvHD on high-dose corticosteroids), who continued with the prophylaxis until at least day +200 (19, 24).

During monitoring, HCMV blips were defined as the detection of HCMV DNA in blood at levels below 10,000 UI/mL, with subsequent spontaneous clearance without requiring antiviral treatment, as previously described (25). Accordingly, clinically significant HCMV infection was defined as the detection of HCMV DNA above the “blip threshold” at one or two consecutive time points during follow-up requiring PET. In such cases, valganciclovir was used as first-line therapy until HCMV DNA became undetectable, with clearance confirmed in two consecutive samples.

HCMV end-organ disease (EOD) was defined as the presence of clinical symptoms and signs of disease in a specific organ in which evidence of HCMV is demonstrated beyond merely detection of CMV in blood and by appropriate diagnostic testing (e.g. by histopathology, immunohistochemistry, or detection of viral antigen or DNA in site-specific fluid or tissue) (26). HCMV EOD was treated with prolonged antiviral therapy, such as valganciclovir, ganciclovir or foscarnet, according to international (27) and internal guidelines.

### 2.3 Other definitions

The intensity of conditioning was defined as previously described (28–30). Rabbit ATG was administered at a total dose of 30 mg/kg, divided over 3 consecutive days prior to stem cell infusion, in recipients of matched sibling donors at high risk of developing GvHD or of 10/10 matched unrelated donor (MUD) allo-SCT mainly following reduced-intensity conditioning (RIC). A total ATG dose of 60 mg/kg was used for recipients of unrelated donor allo-SCT (both matched and mismatched), as previously described (31–33). Neutrophil engraftment was defined as the first of three consecutive days with a neutrophil count ≥ 0.5 x 109/L. Diagnosis and grading of aGVHD followed the 1994 Consensus Conference on Acute GVHD Grading (34), while diagnosis and grading of cGVHD were performed according to NIH Consensus Criteria (35, 36). The European Bone Marrow Transplantation (EBMT)-risk score was calculated as previously described (37).

### 2.4 Monitoring of immune reconstitution

Immune monitoring to assess IR was performed with a flow cytometry using the BD Multitest™ 6-color TBNK reagent with BD TrucountTM tubes on a FACS CantoTM to identify and determine the percentages and absolute counts of T (CD3+), B (CD19+), and natural killer (NK) cells (CD16+CD56+) as well as the CD4 and CD8 subpopulations of T cells in peripheral blood. The immune monitoring was performed every month during the first year after allo-SCT and afterwards every 3 months until IR, which was defined as the detection in 2 consecutive measurements of a CD3+CD4+-T-cells value > 200 cells/µl and a CD19+-B-cells value of > 50cells/µl (38, 39). In the present analysis both time-point of IR and as its kinetics with qualitative and quantitative assessments of the different cell populations on day +30, +100, +180 and +365 were evaluated.

### 2.5 Statistical analysis

Descriptive statistics for continuous variables were reported as medians and ranges. Categorical variables were reported as counts and percentages. Two-group comparisons were performed using Wilcoxon rank sum test and Fisher’s exact test. Descriptive survival analyses were performed using the Kaplan–Meier method. The follow-up distribution was calculated by reverse Kaplan-Meier estimates. Cumulative incidences were calculated via Aalen-Johanson estimator and compared formally between groups via a (propensity score weighted) Fine and Gray model. The propensity score of a patient for LET treatment was calculated using a logistic regression model including the variables age, sex, HCMV serology of donor, disease status at transplant. Donor type was included as a stratification factor. Results of the propensity score model are presented as odds ratios (ORs) with 95% confidence intervals (CIs) and p-values (**Supplementary Table 1**). The distribution and overlap of propensity scores between the two cohorts are shown in **Supplementary Figure 1**. Propensity-score weighted cause-specific Cox proportional hazards models were then used to evaluate the association between LET prophylaxis and the occurrence of IR, considering death without prior IR as a competing event. The multivariable analysis (MVA) was adjusted for relevant clinical variables at baseline, including age at transplantation, sex, donor HCMV serostatus, disease status at transplantation, and conditioning regimen intensity, as well as time-dependent variables such as acute GvHD grade III-IV, chronic GvHD and clinically significant HCMV infections. Donor type was included as stratification factor in the Cox models. Results are presented as cause-specific hazard ratios (HRs) with 95% CIs and p-values. An HR >1 indicates a higher hazard of IR, whereas an HR <1 indicates a lower probability of IR over time. All analyses were performed using R version 4.4.3.

## 3 Results

### 3.1 Cohort description

A total of 276 HCMV seropositive patients undergoing allo-SCT between 01/2013 and 03/2023 were evaluated. Ninety-nine (36%) were in the LET cohort and 177 (64%) were in the PET cohort. The two cohorts were comparable in terms of age at transplantation, underlying hematological disease, disease status at transplant, donor source, and conditioning regimen. There were fewer males (n=49, 49%) in the LET cohort as in the PET cohort (n=113, 64%), p = 0.022. In the LET cohort 66 donors (67%) were HCMV-seropositive versus 151 (85%) in the PET cohort (p<0.001).Patients’ characteristics are summarized in **Table 1**. The median duration of administration of LET in the LET cohort was 147 days (range, 25–1141 days). In 17 patients (17.2%) LET was re-assumed as a secondary prophylaxis due the HCMV clinically significant infection in the context of an active and therapy requiring acute or chronic GvHD. This management strategy ensured a high cumulative exposure to LET, with more than 50% of patients receiving the drug for over 140 days. The cumulative incidence of acute GvHD (any grade) at day +100 was not statistically significant different between the two cohorts (76% [95% CI, 66%-83%] in the LET cohort versus 59% [95% CI, 52%-66%] in the PET cohort, p= 0.173). The same applied for therapy-requiring severe (grade III-IV overall) aGvHD, with a cumulative incidence by day +100 of 26% (95% CI, 17%-34%) in the LET cohort versus 13% (95% CI, 9%-18%) in the PET cohort, p=0.166. Considering the cumulative incidence of cGvHD at 1 year after transplantation, we observed an increase in the occurrence of chronic GvHD in patients receiving LET prophylaxis as compared to the historical cohort (41% [95% CI, 31%-50%] versus 32% [95% CI, 25%-39%], p= 0.007), which differs from data emerging from retrospective studies considering a CNI-free, PT-Cy-based GvHD prophylaxis backbone (21, 40).

**Table 1:** Patients’ and transplantations’ characteristics.

Variable	Values	Total	LET cohort	PET cohort	P value
Number	Number, n (%)	276	99	177	
Age at allo-SCT	Years, median (range)	59 (19-74)	59 (21-72)	59 (19-74)	0.3
Sex	Female, n (%)	114 (41%)	50 (51%)	64 (36%)	**0.022**
	Male, n (%)	162 (59%)	49 (49%)	113 (64%)	
Disease type	AML, n (%)	126 (46%)	47 (47%)	79 (45%)	0.4
	MDS, n (%)	41 (15%)	12 (12%)	29 (16%)	
	MPN, n (%)	41 (15%)	16 (16%)	25 (14%)	
	ALL, n (%)	18 (6.5%)	8 (8.1%)	10 (5.6%)	
	MM, n (%)	8 (2.9%)	0 (0%)	8 (4.5%)	
	NHL, n (%)	30 (11%)	11 (11%)	19 (11%)	
	SAA, n (%)	8 (2.9%)	3 (2%)	5 (2.8%)	
	Other, n (%)	4 (1.4%)	2 (2%)	2 (1.1%)	
Disease status at allo-SCT	Early (CR + upfront), n (%)	176 (64%)	68 (69%)	108 (61%)	0.4
	PR, n (%)	23 (8%)	8 (8%)	15 (8%)	
	PD/REL, n (%)	77 (28%)	23 (23%)	54 (31%)	
Donor type	SIB, n (%)	33 (12%)	5 (5%)	28 (16%)	**<0.02**
	MUD 10/10, n (%)	183 (66%)	69 (70%)	114 (64%)	
	MUD 9/10, n (%)	60 (22%)	25 (25%)	35 (20%)	
Donor source	PBSC, n (%)	270 (98%)	98 (99%)	172 (97%)	0.4
	BM, n (%)	6 (2%)	1 (1%)	5 (3%)	
Conditioning regimen	MAC, n (%)	51 (18%)	19 (19%)	32 (18%)	0.4
	RTC, n (%)	88 (32%)	36 (36%)	52 (29%)	
	RIC, n (%)	137 (50%)	44 (44%)	93 (53%)	
HCMV status patient/donor	Pos/pos, n (%)	217 (79%)	66 (67%)	151 (85%)	**<0.001**
	Pos/neg, n (%)	59 (21%)	33 (33%)	26 (15%)	

LET, letermovir; PET, pre-emptive treatment; allo-SCT, allogeneic stem cell transplantation; AML, acute myeloid leukemia; MDS, myelodysplastic syndrome; MPN, myeloproliferative neoplasm; ALL, acute lymphoblastic leukemia; MM, multiple myeloma; NHL, non Hodgkin lymphoma; SAA, severe aplastic anemia; CR, complete remission; PR, partial remission; PD, progressive disease; REL, relapse; SIB, sibling donor; MUD, matched unrelated donor; PBSC, peripheral blood stem cells; BM, bone marrow; MAC, myeloablative conditioning; RTC, reduced toxicity conditioning; RIC, reduced intensity conditioning; HCMV, human cytomegalovirus.The p value is bold in case it is statistically significant.

### 3.2 Clinically significant HCMV infections

**Figure 1:**
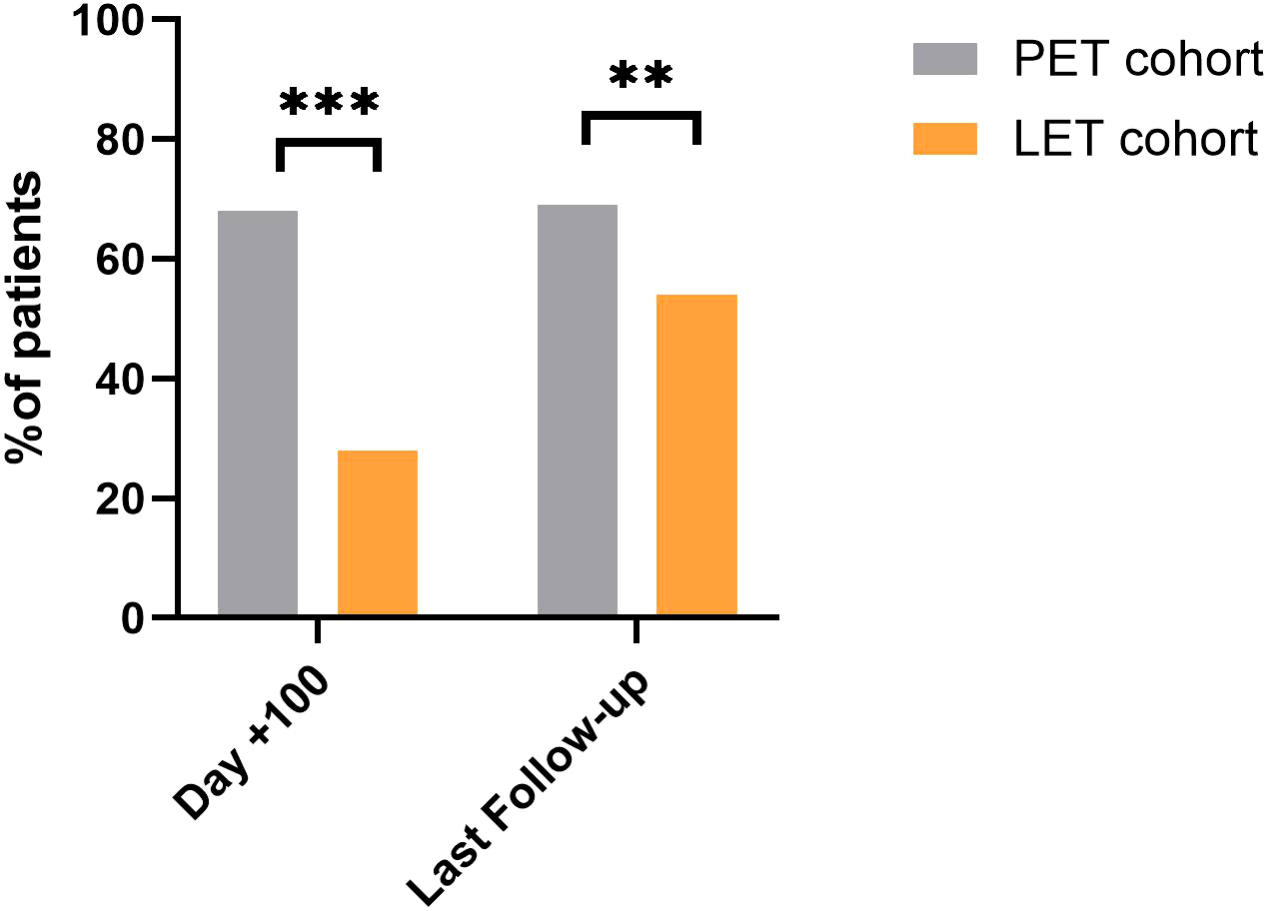
Incidence of clinically significant HCMV reactivations in the letermovir (LET) cohort and in the pre-emptive therapy (PET) historical cohort. Comparison of the percentage of patients developing clinically significant Human Cytomegalovirus (HCMV) reactivation between the letermovir (LET) prophylaxis cohort (orange bars) and the pre-emptive therapy (PET) historical cohort (grey bars). Data are shown at two specific time points: 100 days post-allogeneic stem cell transplantation (Day +100) and at the last available follow-up. ** p≤ 0.01, *** p≤ 0.001.

A total of 205 patients (74%) experienced at least one HCMV reactivation during post–allo-SCT follow-up. As previously reported (18, 19), patients receiving LET had a significantly lower incidence of HCMV reactivation, during the first 100 days after allo-SCT, particularly clinically significant HCMV infections (28 patients (28.3%) in the LET cohort vs. 121 (68.4%) in the PET cohort, p<0.001). This difference was also confirmed analyzing the number of patients developing a clinically significant HCMV infection at the last time-point of follow-up (53 patients (54%) in the LET cohort vs. 123 (69%) in the PET cohort, p=0.009). Data are represented in **Figure 1**. No significant differences were observed between the two cohorts considering the incidence of HCMV blips (12 patients (12%) in the LET cohort vs. 13 patients (7.3%) in the PET cohort, p=0.2. Overall, 46% of the patients in the PET cohort (n=81, 51%) experienced more than one reactivation during follow-up, compared with 36 patients (36%) in the LET cohort (p= 0.4). End-organ disease was documented in 2 patients in the PET cohort (1.1%) and 2 patients in the LET cohort (2%), p= 0.6.

### 3.3 Occurrence of immune reconstitution

**Figure 2:**
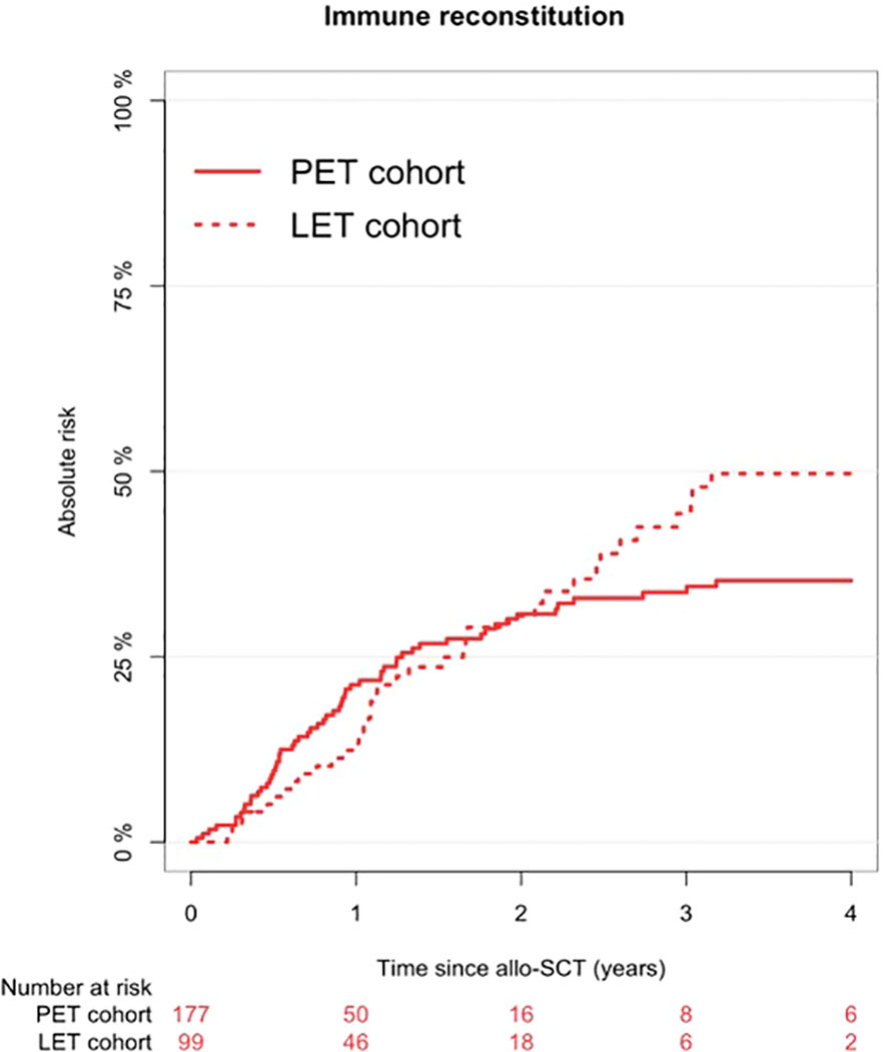
Cumulative incidence of immune reconstitution (IR) in the letermovir (LET) cohort and in the pre-emptive therapy (PET) historical cohort. The cumulative incidence of IR in the LET cohort (dashed line) was 50% (95% CI, 38%-60%) at 4 years after allo-SCT, versus 35% (95% CI, 28%-43%) in the PET cohort (continuous line). This difference between cumulative incidences over the entire follow-up was not statistically significant (p = 0.393). The x-axis shows the time since allogeneic stem cell transplantation (allo-SCT), expressed in years, while the y-axis represents the cumulative incidence (absolute risk, %) of immune reconstitution over time. Solid and dashed step curves depict the cumulative incidence estimates for the PET and LET cohorts, respectively. The table below the plot reports the number of patients at risk at the selected time points (0, 1, 2, 3, and 4 years after allo-SCT) for each cohort, indicating the number of individuals still under observation and event-free at each time point.

After a median follow-up of 4.18 years, a total of 99 patients achieved IR after allo-SCT, with a cumulative incidence of IR at 18 months after allo-SCT of 26% (95% CI, 21%-31%). At this time point we observed a cumulative incidence of IR of 24% (95% CI 16%-33%) in the LET cohort, which was not statistically inferior to what observed in the PET cohort (27% [95% CI 20%-34%], p=0.393). The same applied also considering later time-points of follow-up (**Figure 2**). To account for potential confounding factors, we subsequently assessed the impact of LET on polyclonal IR with a MVA. In this context, LET prophylaxis did not significantly influence the occurrence of IR (HR 1.2, 95% CI 0.74–1.95, p = 0.46). Age at transplant and sex were not significant predictors, even if a not statistically significant trend to a faster IR could be observed for male patients (HR 1.47 for males vs females, 95% CI 0.94–2.29, p = 0.091). Donor HCMV seropositivity was associated with a higher probability of IR (HR 1.81, 95% CI 1.03–3.18, p = 0.039). In addition, patients transplanted in PR showed a significantly higher likelihood of IR compared with those transplanted in CR or upfront (HR 2.98, 95% CI 1.48–5.97, p = 0.002). Among the other variables included in the model, a trend toward reduced IR was observed in patients developing grade 3–4 aGvHD (HR 0.54, 95% CI 0.26–1.12, p = 0.097). Conversely, conditioning intensity showed a non-significant trend toward higher IR in patients receiving RIC conditioning compared with MAC (HR 1.86, 95% CI 0.96–3.59, p = 0.067), whereas no significant difference was observed for the RTC regimen. The results of the MVA are reported in **Table 2**.

**Table 2:** Multivariable analysis of factors associated with immune reconstitution.

Variable	HR	95% CI	p value
Letermovir prophylaxis: No (PET cohort - Ref) Yes (LET cohort)	1.20	0.74-1.95	0.463
Age at allo-SCT	1.00	0.98-1.02	0.990
Patient’s sex: Female (Ref) Male	1.47	0.94-2.29	0.091
HCMV positive donor	1.81	1.03-3.18	**0.039**
Disease status at allo-SCT: CR + upfront (Ref) PR PD/REL	2.98 1.43	1.48-5.97 0.85-2.39	**0.002** 0.177
Conditioning regimen: MAC (Ref) RTC RIC	1.48 1.86	0.70-3.13 0.96-3.59	0.306 0.066
aGvHD grade III-IV*	0.54	0.26-1.12	0.096
cGvHD*	0.63	0.35-1.13	0.123
Clinically significant HCMV infections*	1.44	0.91-2.29	0.116

PET, pre-emptive treatment; LET, letermovir; Ref, reference variable; allo-SCT, allogeneic stem cell transplantation; HCMV, human cytomegalovirus; CR, complete remission; PR, partial remission; PD, progressive disease; REL, relapse; MAC, myeloablative conditioning; RTC, reduced toxicity conditioning; RIC, reduced intensity conditioning; GvHD, graft-versus-host disease; aGvHD, acute GvHD; cGvhD, chronic GvHD.

*time-dependent variables.The p value is bold in case it is statistically significant.

### 3.4 Dynamics of immune reconstitution

**Figure 3:**
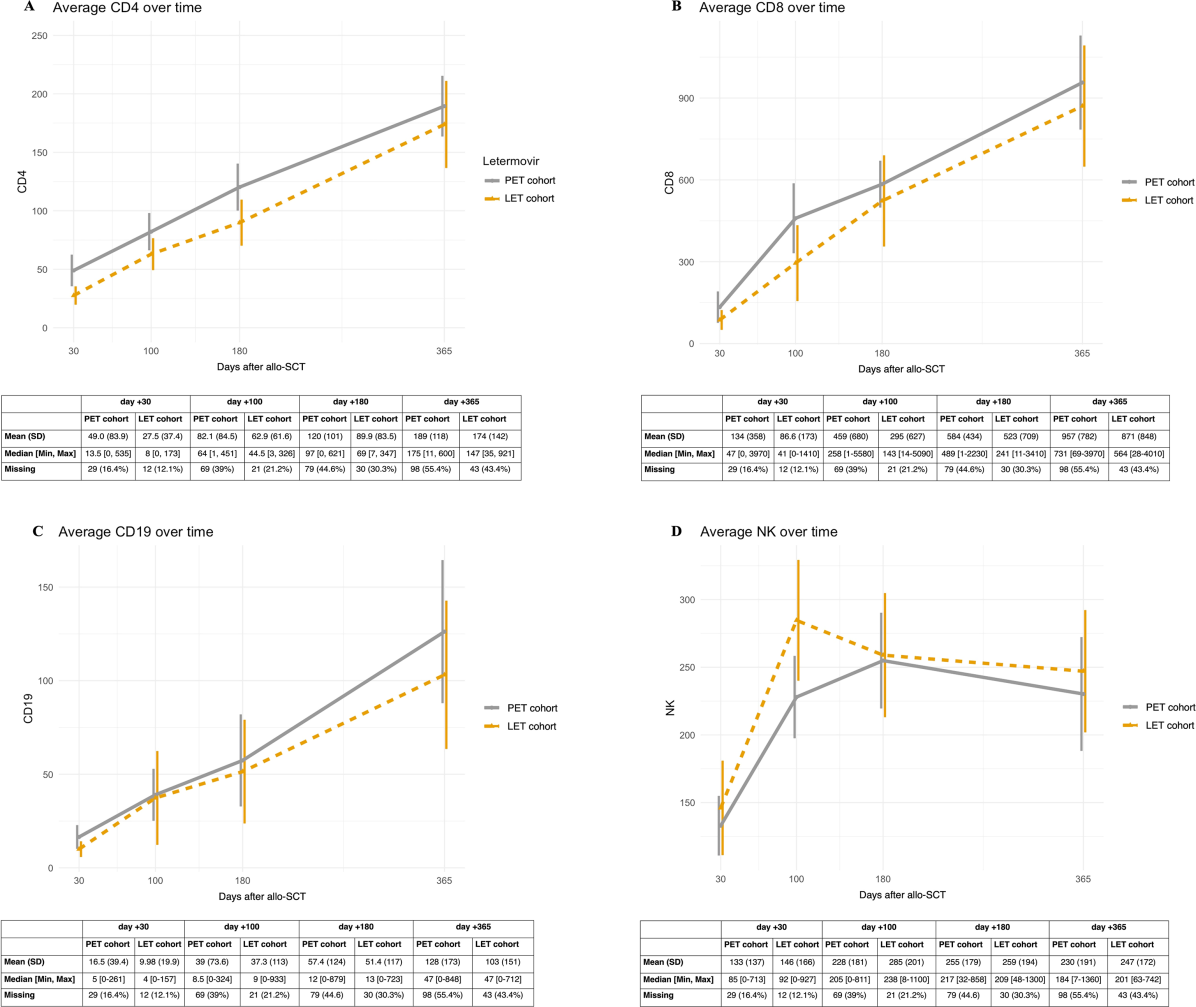
Dynamics of polyclonal immune reconstitution (IR). Cellular sub-populations absolute values are expressed in cells/µl, time after allo-SCT is expressed in days. The absolute counts of cell subpopulations at the evaluated time points (day +30, day +100, day +180, day +365) are reported under each graphic with mean and median values. **(A)** Dynamics of CD3+CD4+ T-cells within the first year after allo-SCT, with letermovir (LET) cohort (dashed yellow line) and pre-emptive therapy (PET) cohort (continuous grey line) showing similar trends and absolute values over time. **(B)** Dynamics of CD3+CD8+ T-cells within the first year after allo-SCT. In the LET cohort we have over time lower absolute values as compared to the PET cohort. **(C)** Dynamics of CD19+ B-cells within the first year after allo-SCT. LET cohort und PET cohort show similar trends and absolute values over time. **(D)** Dynamics of CD16+CD56+ NK cells within the first year after allo-SCT. In the LET cohort an earlier NK cells recovery (day+100) was observed, while in the PET cohort the peak was reached 6 months after allo-SCT.

Given that LET had no significant effect on the occurrence of IR, we investigated whether the temporal dynamics of polyclonal IR differed between patients who received LET for HCMV prophylaxis -experiencing a significantly lower incidence of clinically significant HCMV infections - and the historical PET cohort. Lymphocyte subset counts were evaluated longitudinally at days +30, +100, +180, and +360 post-allo-SCT (**Figures 3A–D**). Overall, all major lymphocyte populations exhibited progressive recovery over time. Focusing on the LET cohort, CD3+CD8+ T cells showed a relatively rapid increase, with median counts rising from 41 cells/µL at day +30 after transplant to 564 cells/µL at day +365. The absolute counts of CD3+CD8+ T cells were lower in the LET cohort as compared to the PET cohort (respectively, 47 cells/µl at day +30 and 731 cells/µl at day +360). CD3+CD4+ T cells expanded more slowly, increasing from 8 cells/µL at day +30 to 147 cells/µL at day +365. B-cell (CD19+) recovery was gradual, with median counts rising from 4 cells/µL at day +30 to 47.0 cells/µL at day +365, whereas NK cells recovered earliest, achieving median levels comparable with those of healthy controls at day +100 after allo-SCT and remaining relatively stable thereafter. Patients in the PET cohort showed CD3+CD8+ T cell counts generally higher at most time points (**Figures 3A, B**). Furthermore, in this cohort, the NK cell expansion tended to occur later, reaching peak levels around day +180. Finally, CD3+CD4+ T cells and B-cell recovery patterns and cell counts were broadly similar between groups. Across all subsets and time points, variability was substantial, as reflected by the wide ranges of observed counts. Despite these descriptive trends, no relevant differences were detected between groups, indicating that LET prophylaxis did not substantially alter the overall kinetics of polyclonal IR in this population.

## 4 Discussion

This is one of the largest retrospective studies evaluating the dynamics and determinants of polyclonal IR after allo-SCT in patients receiving LET for HCMV prophylaxis in the context of an ATG-based GvHD prophylaxis backbone. In our cohort, we confirmed that LET significantly reduces the incidence of clinically significant HCMV infections during the first 100 days post allo-SCT, as previously reported (18, 19). It should be noted that our observed rate of clinically significant HCMV infections (28.3% at day +100 and 54% at the last follow-up) is higher than what was reported in the pivotal phase 3 trial (18). However, this difference could be explained by the high-risk profile of our real-world cohort. Specifically, while 37.5% of patients in the Marty et al. trial received ATG-based GvHD prophylaxis, 100% of our population underwent T-cell depletion with ATG. This induces a deeper and more sustained lymphopenia, significantly slowing immune recovery during post-transplant follow-up, thus increasing the intrinsic risk of HCMV infections. With respect to polyclonal IR, both univariable and multivariable analyses demonstrated that LET does not significantly affect the development or timing of IR after transplantation as compared to our historical cohort (PET cohort), with patients not receiving HCMV prophylaxis with LET. Nevertheless, we observed distinctive immune cell dynamics in the LET cohort compared with the PET cohort. Specifically, in the LET cohort we noted: (1) a trend toward lower absolute CD3+CD8+ T cell counts with a rapid expansion in the early post-transplant months, and (2) an earlier peak of NK cell expansion (around day +100 versus day +180 in the PET cohort). The kinetics of CD3+CD4+ T cells and CD19+ B cells were largely comparable between cohorts. The lower absolute counts of CD3+CD8+ T cells, together with the earlier NK cell peak may be explained by the reduced HCMV exposure in the first post-transplant months, which potentially results in diminished immune stimulation. To our knowledge, this represents one of the largest analyses of the timing and dynamics of polyclonal IR under LET prophylaxis in the context of an ATG-based GvHD prophylaxis backbone. Orofino and colleagues (21) examined cellular and humoral IR in the setting of a PT-Cy-based GvHD prophylaxis, which impacts immune recovery differently than ATG, typically leading to slower IR in the early months post allo-SCT (41, 42). Nevertheless, Orofino et al. confirmed an earlier NK cell expansion peak, together with an improved B-cell reconstitution, which was not observed in our cohort. This difference may reflect the distinct mechanisms of action of ATG and PT-Cy, as ATG mediates direct and prolonged T and B cell depletion, whereas PT-Cy primarily eliminates proliferating alloreactive T cells (43). Similarly, Lauruschkat and colleagues (44) reported comparable findings to ours in a smaller cohort (24 patients in the LET group vs. 32 in the PET group), the majority of whom received ATG-based GvHD prophylaxis. Recently, the impact of LET in the occurrence of IR was assessed in the pediatric population (45). Also in this context LET did not influence the immune recovery after allo-SCT, while markedly reducing the incidence of clinically significant HCMV infections. Importantly, the central message remains consistent across studies: LET does not exert a significant long-term effect on polyclonal IR, while variations in immune recovery trajectories, which at least in part may reflect the influence of the underlying GvHD prophylaxis strategy (PT-Cy versus ATG) in combination with LET.

Our study has some limitations that should be acknowledged. First, its retrospective design with the use of an historical comparison cohort and the lack of a randomized data, which may introduce a bias, even if propensity score analysis demonstrated good balance between the two cohorts. Furthermore, IR was assessed exclusively by peripheral blood counts, without evaluation of functional recovery, antigen specificity (e.g., HCMV-specific T-cells) or detailed immunophenotypic characterization beyond absolute CD4^+^ and CD8^+^ T-cell counts; therefore, it may not fully capture the qualitative aspects of immune recovery. In addition, absolute cell count values at the different follow-up time points could not be statistically compared as the small sample size did not allow longitudinal analyses accounting for potential confounding factors. Consequently, this part of the results should be interpreted as purely descriptive. Moreover, virological and immunological data were collected asynchronously, as HCMV-DNA was monitored weekly while immune subsets were assessed at fixed monthly time points. This precluded robust temporal correlation analyses between viral load, reactivation episodes, and IR. Despite these limitations, our study presents notable strengths. The cohort is large and robust, including 276 consecutive HCMV seropositive adult patients undergoing longitudinal follow-up for one year post-transplant. The analysis performed with the use of propensity score adjustment and multivariable analyses enhance the robustness of our findings by accounting for potential confounders. Finally, a key feature and a novelty aspect of our study is the focus on patients receiving ATG-based GvHD prophylaxis, a setting less extensively characterized in the context of letermovir prophylaxis compared to PTCy platform (21). In this context, while it is essential to identify which patients have developed sufficient immunity to control HCMV reactivation or clinically significant HCMV infection especially after discontinuation of letermovir -and efforts are ongoing in this direction (46) - our results provide evidence for the robustness of global polyclonal IR during exposure to letermovir in the post-ATG lymphodepleted setting.

In conclusion, we demonstrate that LET significantly reduces clinically significant HCMV infections without impairing polyclonal IR. Of note, NK cells exhibited an earlier expansion peak in the LET cohort, suggesting a subtle effect on immune dynamics, although without long-term impact on overall immune recovery. The differences observed compared with prior reports by Orofino and colleagues underscore the importance of the underlying GvHD prophylaxis platform, as ATG- and PT-Cy–based regimens differentially shape IR trajectories. Taken together, our findings support the safe use of LET in the setting of ATG-based GvHD prophylaxis, ensuring effective HCMV prevention while preserving robust IR.

## Data Availability

The data analyzed in this study is subject to the following licenses/restrictions: The dataset is available from the corresponding author upon request. Requests to access these datasets should be directed to elisa.sala@uniklinik-ulm.de.
