# Supplementary material for: Letermovir does not affect long-term polyclonal immune reconstitution after allogeneic hematopoietic stem cell transplantation with ATG-based GvHD prophylaxis
Source: Front Immunol. 2026 May 21;17:1790563. doi: 10.3389/fimmu.2026.1790563 (PMC13233191; doi:10.3389/fimmu.2026.1790563)
Supplement: Supplementary file 1 [file DataSheet1.docx]

***Supplementary Materials***

Letermovir does not affect polyclonal immune reconstitution after allogeneic hematopoietic stem cell transplantation with ATG-based GvHD prophylaxis.

Conrad Weidt^1^, Adela Maria Neagoie^1^, Maral Saadati^2^, Daniel Fürst^3^, Verena Wais^1^, Christian Sinzger^4^, Katrin Strauss^1^, Katharina Göhring^4^, Jacqueline Schnell^1^, Michaela Feuring^1^, Frank Stegelmann^1^, Axel Benner^2^, Detlef Michel^4^, Thomas Stamminger^4^, Hartmut Döhner^1^, Donald, Bunjes^1^, Elisa Sala^1*^

This Supplementary Material provides additional methodological details, figures, and tables supporting the results presented in the main manuscript.

**Supplementary Tables**

**Table S1. Propensity score model for letermovir prophylaxis.**  The propensity score for receiving LET prophylaxis was estimated using a logistic regression model including age at transplantation, sex, donor HCMV serostatus, and disease status at transplantation. Donor type was included as stratification factors. Results are presented as odds ratios (ORs) with 95% confidence intervals (CIs) and corresponding p-values. A p value ≤0.05 was considered statistically significant.

| **Variable** | **Odds Ratio** | **95% CI** | **p value** |
| --- | --- | --- | --- |
| **Age at allo-SCT** | 0.98 | 0.96-1.00 | **0.0389** |
| **Sex** |  |  |  |
| female | Ref |  |  |
| male | 0.53 | 0.31-0.91 | **0.0202** |
| **HCMV serostatus donor** | 0.25 | 0.13-0.47 | **<0.001** |
| **Disease status at allo-SCT** |  |  |  |
| early (CR + upfront) | Ref |  |  |
| PR | 0.72 | 0.27-1.91 | 0.5046 |
| REL/REF | 0.62 | 0.33-1.15 | 0.1269 |

**Abbreviations**: CI= confidence interval; allo-SCT= allogeneic stem cell transplantation; Ref= reference; HCMV= human cytomegalovirus; CR= complete remission; PR= partial remission; REL= relapsed; REF= refractory.

**Supplementary Figures**

**Figure S1.** Density plot showing the distribution of estimated propensity scores, representing the probability of receiving LET prophylaxis, in the LET cohort and the PET cohort. The propensity score was estimated using a logistic regression model including age at transplantation, sex, donor HCMV serostatus, and disease status at transplantation, with donor type included as stratification factor. The overlap between the two distributions indicates comparability between the cohorts and supports the use of propensity score weighting in subsequent analyses.

**
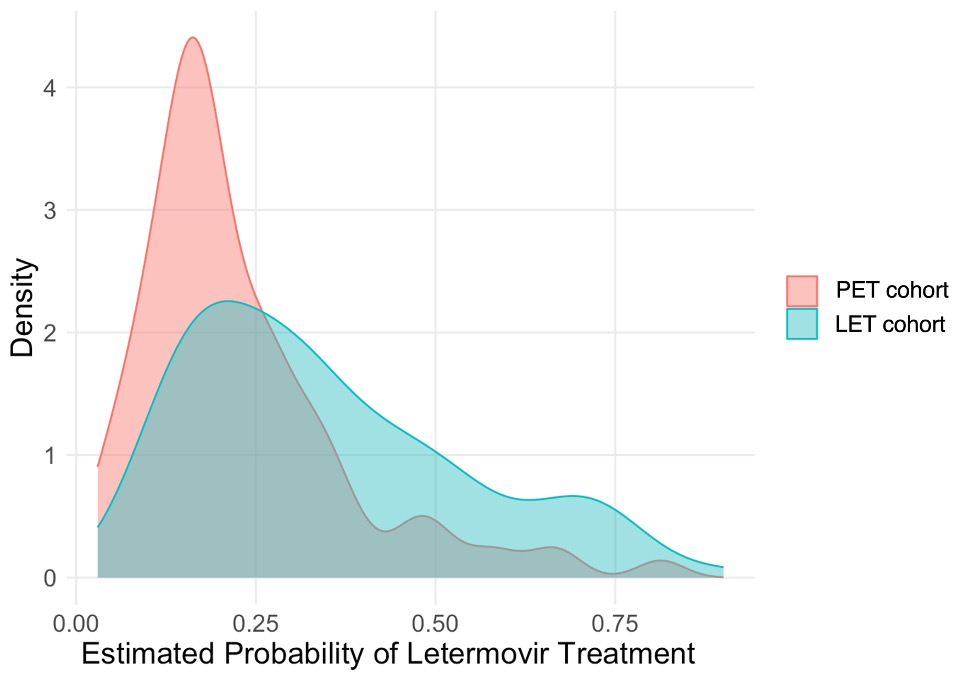
**

**Abbreviations**: PET= pre-emptive treatment; LET= letermovir.
